# Reply to Choi-Kain and colleagues’: Correspondence to Setkowski and colleagues on best psychotherapies for borderline personality disorder

**DOI:** 10.1017/S0033291724002708

**Published:** 2025-02-11

**Authors:** Kim Setkowski, Arnoud Arntz, Pim Cuijpers

**Affiliations:** 1 113 Suicide Prevention, Amsterdam, The Netherlands; 2 Department of Pedagogical and Educational Sciences, University of Groningen, Groningen, The Netherlands; 3 Department of Clinical Psychology, University of Amsterdam, Amsterdam, The Netherlands; 4 Department of Clinical, Neuro and Developmental Psychology, Amsterdam Public Health Research Institute, VU University, Amsterdam, The Netherlands

**Keywords:** borderline personality disorder, suicidal behavior, psychotherapy, network meta-analysis, mental health care

Authors’ reply to Choi-Kain et al. (2023):

We appreciate the judicious comments of Choi-Kain et al. (2023) on our network meta-analysis (NMA) (Setkowski et al., 2023). The authors identified an important challenge within the field, namely that psychological treatments are sometimes labeled differently compared to what they provide. Not all studies included in our NMA provide a clear description of their intervention- and control conditions (e.g., treatment-as-usual). As described in Supplemental Table S2 (Setkowski et al., 2023), some of the included studies provided little to no information about their control conditions, whereas others described them in great detail. However, this is an issue (N)MAs in general face. We therefore encourage the field to be more detailed about the intervention- and control conditions they examine.

The first point the authors address is the classification of general psychiatric management (GPM). We want to thank the authors for critically looking at our article and for pointing out the typo in Table 1. During the process, we considered different categories for GPM, however, after careful consideration, we labeled the treatment as psychodynamic (PDP). We want to emphasize that Table 1 merely provides definitions for the therapies included. The node-making process is depicted in Supplemental Table S2. As described in Supplemental Table S2, GPM was not mentioned as a generic treatment (GT), however, STM, SCM, and CCT (Table 1) were. We perceived GPM as a PDP and analyzed it as such (Table 2). Our methods, results, and conclusions are fully in line with the node-making process. We want to further clarify our reasoning for handling GPM as a PDP. Firstly, McMain et al. (2009) describe in their article that GPM is based on a *‘psychodynamic approach drawn from Gunderson with an emphasis on the relational aspects and early attachment relationships’* and *‘dynamically informed psychotherapy’* (p.1367). This differs from how the authors later describe GPM (labeled ‘Good’ instead of ‘General’), namely as ‘mixed’ and eschewing any focus on early (attachment) relationships (Gunderson et al., 2018). Since we analyzed GPM from McMain’s study, and not its later form, we stand by our decision to classify GPM as a PDP rather than a GT or ‘mixed’. Secondly, the authors argue that GPM overlaps with SCM and STM because these treatments integrate similar guidelines. Even though treatments might be based on the same multidisciplinary guideline, this does not make them similar, as guidelines are often very broadly written and far from specific (Setkowski, 2023).

The second point raised by the authors is related to the classification of manual-assisted cognitive therapy (MACT). We considered therapies to be CBT if they focused on *‘exploring, challenging and restructuring patients’ dysfunctional beliefs’* (Table 1). Weinberg et al. (2006) conducted an RCT to investigate the efficacy of MACT. Another RCT by Evans et al. (1999) describes the chapters of the MACT booklet. We have carefully read Weinberg’s and Evans’ descriptions of MACT. We agree with the authors that MACT contains cognitive-based techniques, such as *‘problem-solving strategies’* (Evans et al., 1999; Weinberg et al., 2006), and *‘self-monitoring of negative thoughts and feelings’* (Evans et al., 1999). However, these cognitive-based techniques do not grasp the core element of CBT. Neither Weinberg et al. (2006) nor Evans et al. (1999) portrayed MACT as an intervention aimed at *‘exploring, challenging and restructuring patients’ dysfunctional beliefs’.* On the contrary, other interventions that we included in our CBT node did explicitly mention this core element. For example, Lin et al. (2019) described that *‘patients work on identifying automatic thoughts, challenging and substituting dysfunctional thoughts with rational thoughts*’ (p.88). This underscores the need for studies to be more detailed about the intervention- and control conditions they investigate.

We classified therapies as ‘mixed’ if they *‘used a combination of multiple techniques from already existing interventions, such as ST, DBT, MBT or PDP’* (Table 1). In contrast to Lin et al. (2019), Weinberg et al. (2006) explicitly mentioned that MACT is ‘*a six session therapy that incorporates elements of DBT, cognitive-behavioral treatment, and bibliotherapy’* (p.483). If therapies are included as ‘mixed’, this does not necessarily mean that we consider them to be an integrated form of therapy. However, they do contain elements from different treatments, making them considerably different from other treatments that do not. The decision to put all these treatments together into one node can result in unwanted heterogeneity.

Finally, the authors argue that the measurement instruments assessing overall BPD severity vary considerably across the included RCTs. This is a well-known issue that has been addressed not only in our NMA but in other studies as well. There are several ways to deal with this issue in (N)MAs. One option is to pool multiple outcomes together into a composite score. For example, if an RCT does not measure overall BPD severity, but does measure single BPD symptoms, this data is pooled into a ‘combined’ effect size. This prevents researchers from completely removing the RCT from their sample and still being able to compute a reliable effect size. Another option is to calculate one effect size per RCT. As we wanted to keep the statistical analyses as simple as possible, we chose the latter option. We performed a sensitivity analysis to check if this decision influenced our results (i.e., only RCTs reporting overall BPD severity were analyzed). It did not.

The point the authors raised about the limited availability of these treatments is exactly what renders the NMA approach useful. We want to emphasize the importance of free availability and further dissemination of treatment manuals, as they present a clear opportunity to not only improve existing guidelines and standards but also to make effective care more available for those who need it (Cuijpers et al., 2024). To prevent substandard delivery of treatments, good manuals should provide information about certain quality criteria, such as the training that clinicians need to have before they can deliver a treatment. We do think patients with BPD can benefit more from treatments such as MBT or DBT if these therapies are based on an even more personalized approach, tailored to their needs.
